# Any physical activity in elderly better than none at all for reducing cardiovascular risk

**Published:** 2018

Any physical activity in the elderly is better than none at all for reducing cardiovascular risk, according to an 18–year study in more than 24 000 adults published recently in the European Journal of Preventive Cardiology. 

‘We know that regular physical activity has major health benefits,’ said first author Dr Sangeeta Lachman, a cardiologist at the Academic Medical Centre, Amsterdam, the Netherlands.

‘Healthy adults are advised to do at least 150 minutes a week of moderate–intensity or 75 minutes a week of vigorousintensity aerobic exercise to reduce their risk of cardiovascular disease,’ she continued. ‘These recommendations are based primarily on research in middle–aged adults and we wanted to know whether regular physical activity yields comparable cardiovascular health benefits in elderly people.’

This study compared the association between different levels of physical activity and the risk of cardiovascular disease in middle–aged to elderly individuals. The hypothesis was that exercise would be equally beneficial in reducing cardiovascular risk in middle–aged and elderly individuals.

The study included 24 502 adults aged 39 to 79 years who participated in the European Prospective Investigation into Cancer (EPIC) Norfolk cohort, a prospective population study that is part of the 10–country collaboration EPIC study. The cohort was primarily designed to assess dietary and other determinants of cancer, but data were also collected on determinants of cardiovascular disease.

Participants were recruited between 1993 and 1997 from registries of general practices in the county of Norfolk, UK. On enrolment into the study, participants completed a health and lifestyle questionnaire, underwent a standardised physical examination and gave blood samples. Physical activity during work and leisure time was assessed with a questionnaire and participants were categorised as active, moderately active, moderately inactive and inactive.

Patients were followed up until 31 March 2015 for hospitalisation or death from cardiovascular events (coronary heart disease or stroke), which were identified by linking the participant’s unique National Health Service number with the East Norfolk Health Authority (ENCORE) database. Physical activity levels and time to cardiovascular events were investigated in three age categories: less than 55, 55–65 (middle–aged), and over 65 years of age (elderly).

During a median follow up of 18 years, there were 5 240 cardiovascular disease events. In elderly participants, hazard ratios for cardiovascular events were 0.86, 0.87 and 0.88 in moderately inactive, moderately active and active people, respectively, compared to inactive people. In those aged 55–65 and less than 55 years, the associations were directionally similar, but not statistically significant.

Dr Lachman said: ‘We observed an inverse association between physical activity and the risk of cardiovascular disease in both elderly and middle–aged people. As expected, there were more cardiovascular events in elderly participants, which could explain why the association only reached significance in this age category.’

‘Elderly people who were moderately inactive had a 14% reduced risk of cardiovascular events compared to those who were completely inactive,’ continued Dr Lachman. ‘This suggests that even modest levels of physical activity are beneficial to heart health. Elderly people should be encouraged to at least do low–intensity physical activities such as walking, gardening and housework.’

She concluded: ‘Given our aging population and the impact of cardiovascular disease on society, a broader array of public health programmes are needed to help elderly people engage in any physical activity of any level and avoid being completely sedentary.’
